# Production of the Invasive Aspergillosis Biomarker Bis(methylthio)gliotoxin Within the Genus *Aspergillus: In Vitro and in Vivo* Metabolite Quantification and Genomic Analysis

**DOI:** 10.3389/fmicb.2018.01246

**Published:** 2018-06-12

**Authors:** Matxalen Vidal-García, Sergio Redrado, M. Pilar Domingo, Patricia Marquina, Cristina Colmenarejo, Jacques F. Meis, Antonio Rezusta, Julian Pardo, Eva M. Galvez

**Affiliations:** ^1^Centro de Investigación Biomédica de Aragón, Instituto de Investigación Sanitaria Aragón, Zaragoza, Spain; ^2^Hospital Universitario Miguel Servet, Zaragoza, Spain; ^3^Instituto de Carboquímica, Zaragoza, Spain; ^4^Department of Medical Microbiology, Radboud University Medical Centre, Nijmegen, Netherlands; ^5^Department of Medical Microbiology and Infectious Diseases, Canisius Wilhelmina Hospital, Nijmegen, Netherlands; ^6^Departamento de Microbiología, Medicina Preventiva y Salud Pública, Universidad de Zaragoza, Zaragoza, Spain; ^7^Fundacion Agencia Aragonesa para la Investigacion y el Desarrollo, Zaragoza, Spain; ^8^Departamento de Bioquímica y Biología Molecular y Celular, Universidad de Zaragoza, Zaragoza, Spain; ^9^Instituto de Nanociencia de Aragón, Zaragoza, Spain

**Keywords:** bis(methylthio)gliotoxin, *Aspergillus* spp., *gtmA*, invasive aspergillosis, biomarker

## Abstract

Gliotoxin (GT) is a fungal secondary metabolite that has attracted great interest due to its high biological activity since it was discovered by the 1930s. An inactive derivative of this molecule, bis(methylthio)gliotoxin (bmGT), has been proposed as an invasive aspergillosis (IA) biomarker. Nevertheless, studies regarding bmGT production among common opportunistic fungi, including the *Aspergillus* genus, are scarce and sometimes discordant. As previously reported, bmGT is produced from GT by a methyl-transferase, named as GtmA, as a negative feedback regulatory system of GT production. In order to analyze the potential of bmGT detection to enable identification of infections caused by different members of the *Aspergillus* genus we have assessed bmGT production within the genus *Aspergillus*, including *A, fumigatus*, *A. niger*, *A. nidulans*, and *A. flavus*, and its correlation with *gtmA* presence. In order to validate the relevance of our *in vitro* findings, we compared bmGT during *in vitro* culture with the presence of bmGT in sera of patients from whom the *Aspergillus* spp. were isolated. Our results indicate that most *A. fumigatus* isolates produce GT and bmGT both *in vitro* and *in vivo*. In contrast, *A. niger* and *A. nidulans* were not able to produce GT or bmGT, although *A. niger* produced bmGT from a exogenous GT source. The frequency and amount of bmGT production in *A. terreus* and *A. flavus* isolates *in vitro* was lower than in *A. fumigatus*. Our results suggest that this defect could be related to the *in vitro* culture conditions, since isolates that did not produce bmGT *in vitro* were able to synthetize it *in vivo*. In summary, our study indicates that bmGT could be very useful to specifically detect the presence of *A. fumigatus*, the most prevalent agent causing IA. Concerning *A. terreus* and *A. flavus* a higher number of analyses from sera from infected patients will be required to reach a useful conclusion.

## Introduction

More than 20 years ago the first invasive aspergillosis (IA) biomarker, galactomannan (GM), was developed based on an enzyme linked immunosorbent assay (Stynen et al., 1995). It stirred up the diagnosis of this lethal infectious disease, as it allowed to detect the infection when combined with clinical signs and symptoms (Maertens et al., 1999, 2002). During the last few years, the biomarker weaponry has arisen with the development of a system to detect β-D-glucan, *Aspergillus* PCR and lateral flow device to detect an *Aspergillus*-derived protein among others (Odabasi et al., 2004; Thornton, 2008; White et al., 2015). New diagnostic approaches were developed based on the increased accuracy of these tests, such as pre-emptive therapy (Wingard, 2007; Riwes and Wingard, 2012). Despite of these advances, IA management continues to be challenging due to the heterogeneous population at risk, the diversity of clinical and radiological presentations and the lack of a gold standard (Lamoth and Calandra, 2017). Thus, at present, it is required to understand the limitations of each biomarker and the corresponding diagnosis test in order to accurately diagnose these challenging infections (Maertens et al., 2016). In this line, the future directions in IA diagnosis research need to focus on the development of new biomarkers, including a clear understanding of their strengths and limitations, along with the assessment of their utility in well-designed clinical trials (Arvanitis and Mylonakis, 2015; Mercier and Maertens, 2017).

In recent years, bis(methylthio)gliotoxin (bmGT) has generated great interest as an IA biomarker (Maertens et al., 2016; Mercier and Maertens, 2017). Its detection in serum by High Performance Thin Layer Chromatography (HPTLC) was shown to be reliable (Domingo et al., 2012). Moreover, it has been clinically validated in a small prospective study in comparison with GM quantification (Vidal-García et al., 2016). Data suggest a good diagnostic performance (61.5% sensitivity and 93% specificity) and importantly, high positive and negative predictive values when used in combination with GM detection (100% and 97.5%, respectively), which suggest a potential utility in pre-emptive approaches. Pending further validation, unlike GM, bmGT detection could be useful in non-immunocompromised populations as it was previously found to be positive in a non-compromised patient suffering from IA that presented negative GM values (Vidal-García et al., 2017). Nevertheless, data regarding the frequency and distribution of bmGT production by different opportunistic molds are scarce and in most cases based on bioinformatics analysis (Bergmann et al., 2007; Andersen et al., 2013; Dolan et al., 2014). These data would be very important for understanding the specificity and the clinical sensitivity of this biomarker to differentiate between species within the *Aspergillus* genus and, thus, treat this infection more effectively.

Bis(methylthio)gliotoxin is an inactive derivative of gliotoxin (GT). *A. fumigatu*s is, to date, the most important opportunistic fungi producing bmGT (Li et al., 2006; Guimarães et al., 2010; Domingo et al., 2012; Sun et al., 2012; Liang et al., 2014). BmGT serves as a negative regulator of the GT biosynthesis, and it is produced by methylation of GT by an S-adenosylmethionine-dependent bis-thiomethyltransferase (Dolan et al., 2014, 2015, 2017), eliminating the ability of GT to produce toxic reactive oxygen species (ROS) (Dolan et al., 2015). BmGT formation from an exogenous source of GT has been described in *A. niger* and *A. nidulans* (Scharf et al., 2014; Manzanares-Miralles et al., 2016). The enzyme responsible for bmGT biosynthesis, which is an *S*-adenosylmethionine (SAM)-dependent methyltransferase called GtmA, has been characterized in *A. fumigatus*; and other orthologs have been found on species like *A. niger* or *A. terreus* (Dolan et al., 2014; Scharf et al., 2014; Manzanares-Miralles et al., 2016). The best characterized enzyme is GtmA, which is known to be encoded by the *gtmA* gene, located in the chromosome 2 (Dolan et al., 2014). Bioinformatics analysis of the *Ascomycota* phylum showed 124 orthologs of GtmA. However, it is known that toxin production is discontinuous among different species and it is not clear which species within *Aspergillus* genus are able to produce GT and, subsequently, the inactive derivative bmGT (Gardiner and Howlett, 2005; Patron et al., 2007). The aim of the present study was to assess the frequency and species distribution of bmGT within the *Aspergillus* genus in cultures *in vitro* as well as *in vivo* in sera of patients from whom fungi were isolated. We also characterized the ability of different clinical isolates from *Aspergillus* genus to methylate GT in cultures *in vitro*, to confirm the presence and activity of methyltransferases in *Aspergillus* species isolated from probable and proven cases of IA. Our findings indicate that bmGT could be considered as an specific biomarker to detect infections by *A. fumigatus*, the most common agent causing IA, excluding the presence of *A. nidulans* and *A. niger*.

## Materials and Methods

### Gliotoxin and Bis(methylthio)gliotoxin Production

We analyzed GT and bmGT production within 252 *Aspergillus* spp. isolates. Most *A. fumigatus* complex (*n* = 119) were clinical isolates from Canisius-Wilhelmina Hospital, Nijmegen (Netherlands). Eighteen of those isolates were cryptic species from Section *Fumigati* from Gregorio Marañón University Hospital, Madrid (Spain). Other *Aspergillus* species were clinical isolates from Miguel Servet University Hospital, Zaragoza (Spain) and corresponded to 36 *A. flavus* complex, 35 *A. terreus* complex, 40 *A. niger* complex, and 22 *A. nidulans* complex. One milliliter of 12 McFarland conidial suspension (approximately 3–5 × 10^7^ conidia/mL) was added to 9 mL of liquid medium (Roswell Park Memorial Institute [RPMI] 1640 + glucose 20 g/L + glutamine 2 mM + HEPES 25 mM) in 50 mL culture flasks and incubated at 37°C for 96 h. A 2 mL sample of supernatant was obtained and frozen at -20°C and subsequently used for GT and bmGT detection and quantification by HPTLC as described below. In those cases where GT and/or bmGT was not detected, fungal isolates were cultured employing Czapek Dox Broth (+ glutamine + HEPES) to confirm that this defect was not specific for RPMI medium. Czapek Dox Broth is a medium of a different composition to RPMI1640, and similarly to the last one, it is commonly used in *Aspergillus* cultures *in vitro.* Thus, we decided to compare both in order to discard effects relative to specific culture media conditions *in vitro*.

### Bis(methylthio)gliotoxin Production From Exogenous Gliotoxin

Bis(methylthio)gliotoxin production from an exogenous source of GT was assessed in a total of 35 isolates of the species complexes *A. flavus* (*n* = 12), *A. terreus* (*n* = 9), *A. niger* (*n* = 8), and *A. nidulans* (*n* = 6). Conidial inoculum was prepared as described above and added to 50 mL culture flasks with Czapek Dox Broth (+ glutamine + HEPES). These cultures were incubated at 37°C for 45 h. At 45 h, GT was added to a final concentration of 2.5 mg/L and methanol was added as solvent control. At 0, 3, and 6 h, 2 mL aliquots of supernatant were taken and frozen until GT and bmGT analysis.

### Detection of Bis(methylthio)gliotoxin in Sera From IA Patients

We assessed sera from patients hospitalized in the Miguel Servet University Hospital with probable/proven IA according to the EORTC/MSG definitions (De Pauw et al., 2008). We included in the study those cases with *Aspergillus* spp. growth in clinical samples. Serum were prospectively collected and frozen at -20°C until GT and bmGT detection. All protocols were supervised and approved by the Ethics Committee of Clinical Research from Aragón (CEICA), number PI15/0203.

### Metabolite Identification and Quantification by High Performance Thin Layer Chromatography (HPTLC)

Gliotoxin and bis(methylthio)gliotoxin detection and quantification were performed both in serum and supernatant samples by HPTLC as described by Domingo et al. (2012). Briefly, GT and bmGT were extracted together using dichloromethane. After agitation and two phase’s separation, non-aqueous phase was added onto silica gel plates. Then, they were developed using a horizontal development chamber (Camag). The mobile phase was a mixture of tetrahydrofuran/*n*-heptane/acetonitrile (40:58:2 [v/v/v]). After 25 min development, plates were scanned with an ultraviolet scanning densitometry (TLC Scanner 3, Camag; λ = 280 and 367 nm; linear scanning). GT and bmGT identification was performed by retention time and spectral analysis and quantification was performed by peak area under curve analysis using Camag’s personal computer software.

### Genetic Detection of *gtmA* Gene

Chromosomal DNA of Aspergillus spp. isolates was extracted using cetyltrimethylammonium bromide (Sigma-Aldrich, St. Louis, MO, United States). The specie-specific primers used for *gtmA* detection are summarized in **Table 1**, along with expected amplicon length. PCR was performed using HotTaq Master Mix, (IBIAN Technologies, Zaragoza, Aragón, Spain). An initial denaturation of 2 min at 94°C was followed by 35 cycles at 94°C for 30 s, 56°C for 15 s, and 72°C for 1 min. DNA amplification products were visualized after electrophoresis on 2% agarose gels. UView 6x loading dye (Bio-Rad, Hercules, CA, United States) was used for nucleic acid staining. As a length standard 0.1–1 kbp molecular mass marker was used.

**Table 1:** Primers.

Name	Sequence (5′–3′)	Amplification length (bp)
*A. terreus* Fw	TCG GAG GCC CTA AAC CG	291
*A. terreus* Rv	GGA TTC GGA AGT CCA ACA AGG	
*A. flavus* Fw	TCA AGC GTC CTT CAT CAT AC	289
*A. flavus* Rv	TCG TCA GGG AAG AGA TTA AAA GC	
*A. niger* Fw	TCT AGT GCC CTT CAT CGT GC	223
*A. niger* Rv	TCG TCA GGG AAG AGG TTG AAC	
*A. fumigatus* Fw	TCC AGC GTA CTC AAC CAC AC	293
*A. fumigatus* Rv	CGT CTG GAA AGA TCT GGA AG	
ITS 1 Fw	TCC GTA GGT GAA CCT GCG G	565 to 613
ITS 4 Rv	TCC TCC GCT TAT TGA TAT G	

### Statistical Analysis

Statistical analyses were performed using Prism 6, Graphpad (San Diego, CA, United States). GT and bmGT concentration in culture filtrates are given as mean ± standard error of mean (SEM). A significance level of 0.05 was considered statistically relevant for multiple comparison test and chi-square test, as appropriate.

## Results

### Gliotoxin and Bis(methylthio)gliotoxin Production by *Aspergillus* spp. Isolates

**FIGURE 1:**
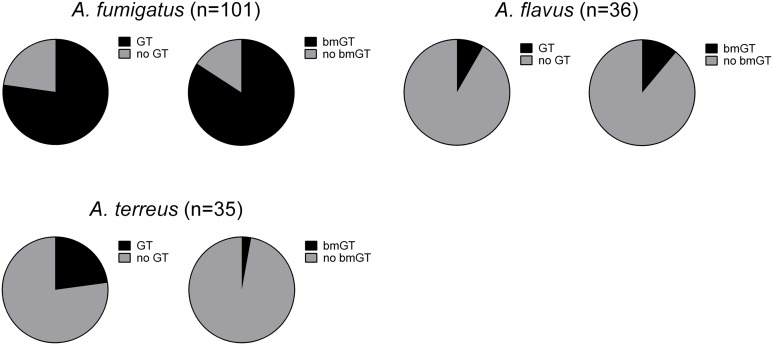
Percentage of gliotoxin and bis(methylthio)gliotoxin producing isolates among different *Aspergillus* species.

The production of GT and bmGT was tested in culture supernatants of 252 *Aspergillus* isolates from the species complexes *A. fumigatus, A. flavus, A. terreus, A. niger*, and *A. nidulans* after 4 days of incubation. Non-cryptic *A. fumigatus* isolates (*n* = 101) produced GT and bmGT at highest frequencies, 77.23% and 84.16%, respectively. Among the five cryptic species analyzed: *A. calidoustus* (*n* = 1)*, A. fumigatiaffinis* (*n* = 3)*, A. lentulus* (*n* = 11)*, N. udagawae* (*n* = 2), and *A. novofumigatus* (*n* = 1), all but the last produced GT and/or bmGT. *A. fumigatiaffinis* seemed to be the most frequent GT and bmGT producing species, as the three tested isolates (100%) produced bmGT. In contrast, just two of the eleven isolates (18%) of *A. lentulus* produced bmGT. BmGT was also more frequently detectable than GT in culture supernatants of *A. flavus*, 11.11% vs. 8.33%, respectively. In contrast, *A. terreus* produced GT more frequently than bmGT (22.86% vs. 2.86% respectively) (**Figure 1**). Notably, none of the *A. niger* and *A. nidulans* isolates tested produced GT and/or bmGT. All cultures were analyzed by HPTLC, a method to detect GT and bmGT previously optimized and validated versus LC-MS (Domingo et al., 2012), and some of the results in selected culture isolates of *A. fumigatus, A. niger, A. nidulans*, and *A. flavus*, were confirmed by LC-MS (data not shown). The absence of GT and/or bmGT production was not specific for the culture conditions *in vitro* since culture of GT/bmGT negative fungal isolates employing Czapek Dox Broth yielded similar results (data not shown). In addition, the differences were not due to different fungal growth since cell cultures showed a similar behavior and growth as analyzed by XTT reduction assay. Optimal culture conditions, as well as analytical specificity of the method, were confirmed by employing cell cultures from an *A. fumigatus gliP* deletion mutant, which is unable to produce GT and bmGT (Sugui et al., 2007).

**FIGURE 2:**
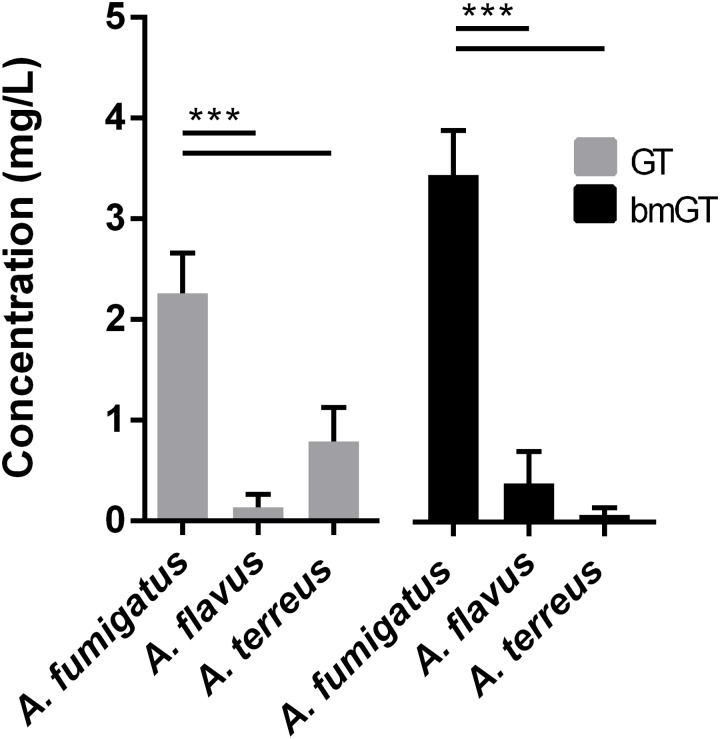
Mean concentrations of gliotoxin and bis(methylthio)gliotoxin detected in culture supernatans of different *Aspergillus* species. ^∗∗∗^Statistically significant differences (*p* < 0.001).

*A. fumigatus* isolates also produced GT and bmGT in higher concentration than other *Aspergillus* spp. did. These differences were statistically significant (*p* < 0.05) (**Figure 2**). The mean concentration of GT was 2.26 ± 0.40 mg/L and of bmGT was 3.45 ± 0.44 mg/L for *A. fumigatus. A. flavus* isolates yielded a mean concentration of 0.14 ± 0.13 mg/L of GT and 0.39 ± 0.31 mg/L of bmGT. The mean concentration of GT and bmGT were 0.79 ± 0.34 mg/L and 0.07 ± 0.07 for *A. terreus*.

### Bis(methylthio)gliotoxin Production From Exogenous Gliotoxin

In order to confirm the results obtained in cell cultures concerning bmGT production, we analyzed the ability of some fungal isolates to generate bmGT from an external GT source as well as the presence of methyltransferase genes. This is of special utility to find out whether the *Aspergillus* spp. that did not produce GT and bmGT (*A. niger* and *A. nidulans*) are also unable to methylate exogenous GT. This finding would mean that these isolates do not express methyl-transferase activity and, thus, confirm that they are unable to generate bmGT. Moreover, this would indirectly suggest that they are also unable to generate GT, since GT methylation has been proposed as a negative feedback regulatory system, inherent to all GT-producing species.

**FIGURE 3:**
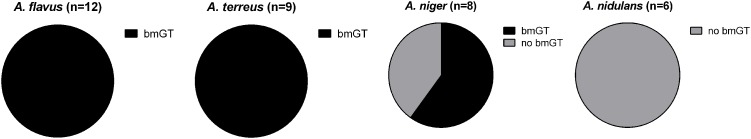
Percentage of GT-methylating isolates of *Aspergillus* species.

First, we analyzed if fungal isolates presented GT methyl-transferase activity by adding pure GT and monitoring the generation of bmGT. The ability to produce bmGT from an exogenous source of GT was assessed in the isolates of *A. flavus, A. terreus, A. niger*, and A. *nidulans* that did not produce GT or bmGT in the previous experiment. All isolates from the *A. flavus* (*n* = 12) and *A. terreus* species (*n* = 9) were able to methylate exogenous GT in order to produce bmGT. Among *A. niger* isolates, this ability was less consistent, nevertheless, 5/8 isolates (62.5%) showed such ability. Finally, none of the *A. nidulans* isolates (*n* = 6) methylated GT to generate bmGT (**Figure 3**). This result confirms that *A. nidulans* does not express methyl-transferase activity and, thus, it is unable to generate endogenously bmGT, and, likely, GT, in line with the results of **Figure 1**. Concerning *A. niger*, some isolates seem to express methyl-transferase activity against exogenous GT. Indeed, it has been previously shown that the methyl-transferase MT-ii is expressed in *A. niger* and methylates exogenous GT (Dolan et al., 2017). However, since they do not produce GT (**Figure 1** and Manzanares-Miralles et al., 2016), this would explain that they are unable to endogenously produce bmGT.

**FIGURE 4:**
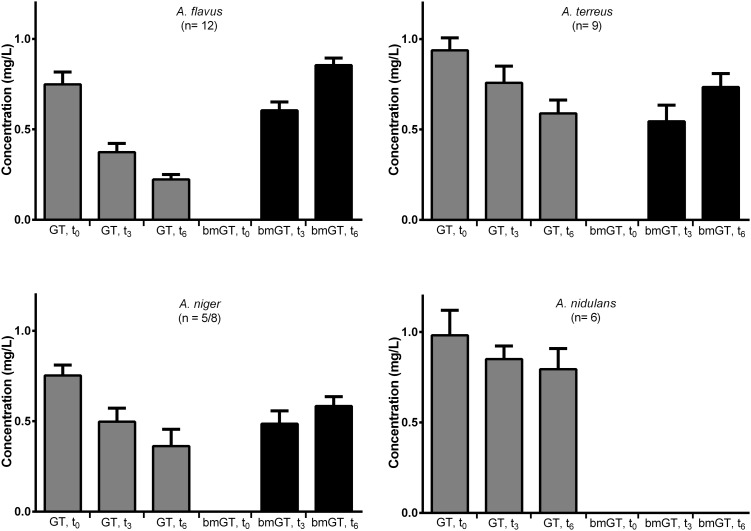
Bis(methylthio)gliotoxin production from exogenous gliotoxin among different *Aspergillus* species. The bars represent mean concentrations with standard error of means.

Among bmGT producing isolates, immediately after GT addition (*t* = 0 h), this was recovered in a mean concentration of 0.86 ± 0.04 mg/L. None of the isolates produced bmGT at this time (**Figure 4**). At 3 h after GT addition, GT concentration decreased and bmGT concentration increased. This observation continued at 6 h, when the maximum bmGT and the minimum GT concentrations were detected. There were no differences between mean concentration of GT and bmGT among species (*p* > 0.05) indicating a similar methylating activity.

### Comparison of Serum bmGT Detection *in Vivo* With bmGT Production in *in Vitro* Cultures

Our results confiults confirm that most *A. fumigatus* isolates and some *A. terreus* and *A. flavus* isolates were able to endogenously and exogenously produce bmGT. However, the frequency of bmGT production within *A. terreus* and *A. flavus* isolates was much less than in *A. fumigatus* isolates. Since the analyses of the genes involved in GT synthesis is difficult due to the complexity of the pathways involved, we decided to analyze if the isolates that did not produce endogenously GT and bmGT *in vitro*, were able to synthesis bmGT in humans *in vivo*. To this aim, we included six cases of probable/proven IA with mycological growth from whom *in vitro* cultures had been established and analyzed. Serum bmGT concentration for these patients, fungal isolation, sample type and fungal ability to produce bmGT *in vitro* (*de novo* and from exogenous GT) as well as detection of *gtmA* gene and the MT-ii homolog are summarized in **Table 2**.

**Table 2:** bmGT production, methylation of exogenous GT and carriage of *gtmA or MT-ii* genes for *Aspergillus* spp. isolates from probable and proven invasive aspergillosis cases.

Case	IA type	Culture (sample)	Serum bmGT (mg/L)	Specie	Supernatant bmGT (mg/L)	GT methylation ([bmGT]_t=6_)	*gtmA* gene
1	Proven	Sinus biopsy	1,66	*A. flavus*	Not detected	0.93	+ (mt-ii)
2	Proven	Vitreous, thrombus	0,19	*A. fumigatus*	0.26 ± 0.05	0.90	+
3	Probable (proven IFI)	Bronchial aspirate	6,84	*A. fumigatus*	0.52 ± 0.05	0.55	+
4	Probable	Sputum	13,68	*A. terreus*	Not detected	0.16	+ (mt-ii)
5	Probable	Bronchial aspirate	–	*A. fumigatus*	0.48 ± 0.14	0.98	+
6	Probable	Sputum	2,6	*A. fumigatus*	0.18 ± 0.02	0.48	+

**FIGURE 5:**
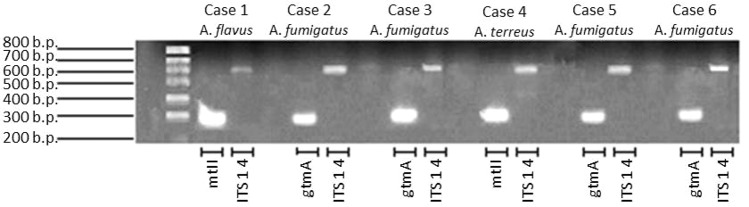
Detection of *gtmA* and mt-ii genes in clinical isolates from probable/proven invasive aspergillosis cases.

All, but one serum, were positive for bmGT. This serum belonged to a patient with probable IA diagnosed by *A. fumigatus* growth in bronchial aspirate. The other three patients with *A. fumigatus* isolation had positive bmGT (range 0.19–13.68 mg/L). There was a case of IA by *A. flavus* and a case of IA by *A. terreus*. Both had detectable bmGT in serum. Notably, these isolates corresponded to those ones in which we were not able to detect either endogenous GT or bmGT during *in vitro* culture. Nevertheless, all of them methylated the exogenous GT and carried the mt-ii methyl-transferase gene, as seen in **Figure 5**. Of note, those isolates which produced bmGT in higher amounts *in vitro*, did not correlate with the highest bmGT production *in vivo*. All the clinical isolates from *A. fumigatus* showed the ability to methylate exogenous GT and carry the *gtmA* gene.

Finally, in order to confirm that *A. nidulans* and *A. niger* did not produce GT and, thus, are unable to endogenously synthesize bmGT, we analyze the presence of *gliP* gene (a critical gene within the gli cluster responsible for GT synthesis) by PCR. None of the isolates from *A. niger* and *A. nidulans* carry the *gliP* gene (data not shown) confirming that they are unable to produce GT and bmGT, as found in the cell culture analysis (**Figure 1**). Moreover, a bioinformatic analyses searching for the presence of gli cluster homology sequences in the genome of sequenced *A. niger* and *A. nidulans* strains, yielded negative results, confirming our experimental data and in line with previous findings (De Pauw et al., 2008; Manzanares-Miralles et al., 2016). In contrast, sequences with some homology to gli cluster were found in both *A. terreus* and *A. flavus* genomes (data not shown) as previously indicated (Patron et al., 2007).

## Discussion

Despite recent advances, the lack of a single gold standard technique and the limitations of the available ones, make diagnosis of IA still challenging (Maertens et al., 2016; Mercier and Maertens, 2017). In recent years, new metabolite based diagnostic tools have been under research, such as GT or volatile organic compounds (Lewis et al., 2005a; Chambers et al., 2009).

Regarding GT, its high biological reactivity and its potential ability to interact with cells and tissues (Domingo et al., 2012) make it hard to detect in body fluids (Scharf et al., 2012). This limitation is overcame by bmGT, which is more stable and reliably detected in serum (Domingo et al., 2012). It is known that *A. fumigatus* produces GT in the highest concentrations and more frequently than other *Aspergillus* species (Lewis et al., 2005b; Kupfahl et al., 2008). This conclusion has been supported by our results, in which 77% of the *A. fumigatus* isolates were GT-producers in significantly higher concentrations and frequencies than those obtained for *A. flavus* and *A. terreus* species complexes, the other *Aspergillus*-producing GT species. With reference to bmGT, *A. fumigatus* was also the most common bmGT producing complex. Notably, and in line with previous findings (De Pauw et al., 2008; Manzanares-Miralles et al., 2016), neither *A. niger* or *A. nidulans* strains were able to endogenously produce GT and/or bmGT, although methyl-transferase activity was found in *A. niger* isolates when using exogenous GT. These results are supported by both PCR analyses and bioinformatic studies confirming the absence of the gli cluster in these species. These findings contrast to previous studies in which a high proportion of *A. niger* isolates were shown to produce GT (Kupfahl et al., 2008). We have no explanation for these contradictory findings although, in line with our findings, Kupfahl et al. (2008) did not detect *gliP* gene in *A. niger*, which has been shown to be critical for GT synthesis, at least in *A. fumigatus* isolates (Sugui et al., 2007).

Some authors have been interested in the secondary metabolite profiles of cryptic species of the Fumigati section. Unlike other authors, we found out that *A. lentulus* and *A. fumigatiaffinis* were able to produce GT and bmGT (Larsen et al., 2007; Sugui et al., 2010; Tamiya et al., 2015). Regrettably, we just analyzed one isolate of *A. calidoustus* and *A. novofumigatus*. The *A. calidoustus* isolate was GT and bmGT producer, but *A. novofumigatus* was not. This result does not rule out the ability to produce GT and bmGT by *A. novofumigatus* since culture conditions (medium, aeration, temperature, sampling time…) affect to secondary metabolite synthesis (Belkacemi et al., 1999; Watanabe et al., 2004). This could explain the low GT and bmGT detection among non-*A. fumigatus* species since all experiments were performed in the same conditions and confirmed employing other culture protocols.

Aiming to avoid such a limitation, we analyzed the ability to produce bmGT from an exogenous source of GT among non-toxigenic isolates. We detected bmGT in culture filtrates of all the *A. flavus* and *A. terreus* analyzed, thus suggesting a consistent ability to produce bmGT. To our knowledge no specific methyltransferases had been described to date for these species. Nevertheless, it has been described an ortholog and a homolog of GtmA for *A. terreus* and *A. flavus* by bioinformatics analysis but it is the first time in which its expression has been described. In our study, *A. niger* also showed the ability to methylate GT, but less frequently. Curiously, none of the *A. nidulans* isolates analyzed produced bmGT even when it has been described that a specific methyltransferase (and the encoding gene) able to produce bmGT from GT for this species (Manzanares-Miralles et al., 2016). This discrepancy could be due to the known fact that culture conditions do not reflect the genetic potential and that not all the strains of the same species have the same metabolic profile (Bergmann et al., 2007). Indeed, secondary metabolism confers a survival benefit to the producing isolate and the *in vitro* culture conditions are not optimal to activate this survival pathway, depending on the *Aspergillus* spp. and/or isolate.

In order to overcome the limitations of the *in vitro* culture to analyze secondary metabolism, we have employed some clinical isolates from patients with probable/proven IA and compared bmGT production in *in vitro* culture with bmGT in serum from those patients. In this scenario, where fungi has to colonize the host and adapt itself to the new environmental conditions, the fungi would activate secondary metabolism and display all potential virulence factors such a GT (Cramer et al., 2006; Sugui et al., 2007) In these conditions, all but one of the seven probable/proven patients had bmGT detectable in serum, even those that did not produce GT and bmGT *in vitro*. Importantly, all of them were able to produce bmGT from exogenous GT and carried an ortholog of GtmA, MT-ii.

In summary, our findings indicate that bmGT production is useful to diagnose IA caused by *A. fumigatus* and, at some extent, by *A. terreus* and *A. flavus*, although at a much lower frequency, since they present the ability to methylate GT and endogenously produce bmGT *in vitro* and *in vivo*. Moreover, and pending of validation with a higher number of samples, our novel findings indicate that conclusions about the expression of molecules that could be used as potential diagnostic biomarkers based on *in vitro* fungal cultures cannot be reached unless they are confirmed in proper *in vivo* studies employing animal models or patients suffering from IA.

## Author Contributions

MV-G and SR carried out the experiments. PM helped with genomic analysis and CC helped with *in vitro* experiments. MD performed the HPTLC analysis. MV-G wrote the manuscript with support from AR, JP, and EG. JM provided and characterized different isolates of *Aspergillus* spp. JP and EG conceived the original idea and supervised the project with the support of AR.

## Conflict of Interest Statement

MD, JP, and EG are co-inventors of a patent licensed to Blackhills Diagnostic Resources S.L. that protects the use of bmGT to diagnose IA (PCT/EP2012/058,247). The remaining authors declare that the research was conducted in the absence of any commercial or financial relationships that could be construed as a potential conflict of interest.
